# Mitral valve orifice area predicts outcome after biventricular repair in patients with hypoplastic left ventricles

**DOI:** 10.1016/j.jocmr.2024.101029

**Published:** 2024-02-23

**Authors:** David Liddle, Addison Gearhart, Lynn A. Sleeper, Minmin Lu, Eric Feins, David N. Schidlow, Sunil Ghelani, Andrew J. Powell, Sitaram Emani, Rebecca S. Beroukhim

**Affiliations:** a The Heart Institute, UPMC Children’s Hospital of Pittsburgh, University of Pittsburgh School of Medicine, Pittsburgh, PA, USA; b Department of Cardiology, Boston Children’s Hospital, Boston, MA, USA; c Department of Pediatrics, Harvard Medical School, Boston, MA, USA; d Department of Cardiac Surgery, Boston Children’s Hospital, Boston, MA, USA; e Department of Surgery, Harvard Medical School, Boston, MA, USA

**Keywords:** Congenital heart disease, Hypoplastic left heart syndrome, Mitral valve, Biventricular

## Abstract

**Background:**

Identification of risk factors for biventricular (BiV) repair in children with hypoplastic left ventricles (HLV) has been challenging. We sought to identify preoperative cardiovascular magnetic resonance (CMR) predictors of outcome in patients with HLVs who underwent BiV repair, with a focus on the mitral valve (MV).

**Methods:**

Single-center retrospective analysis of preoperative CMRs on patients with HLV (≤50 mL/m^2^) and no endocardial fibroelastosis who underwent BiV repair from 2005–2022. CMR measurements included MV orifice area in diastole. The primary composite outcome included time to death, transplant, BiV takedown, heart failure admission, left atrial decompression, or unexpected reoperation; and the secondary outcome included more than or equal to moderate mitral stenosis and/or regurgitation.

**Results:**

Median follow-up was 0.7 (interquartile range 0.1, 2.2) years. Of 122 patients [59 atrioventricular canal (AVC) and 63 non-AVC] age 3 ± 2.8 years at the time of BiV repair, freedom from the primary outcome at 2 years was 53% for AVC and 69% for non-AVC (log rank p = 0.12), and freedom from the secondary outcome at 2 years was 49% for AVC and 79% for non-AVC (log rank p < 0.01). Independent predictors of primary outcome for AVC patients included MV orifice area z-score <−2 and transitional AVC; for non-AVC patients, predictors included MV orifice area z-score <−2, abnormal MV anatomy, and conal-septal ventricular septal defect. Independent predictors of secondary outcome for AVC patients included older age at surgery, transitional AVC, and transposition of the great arteries.

**Conclusion:**

In children with HLV, low MV orifice area and pre-existing MV pathology are risk factors for adverse outcome after BiV repair.

## Graphical abstract

**Figure:**
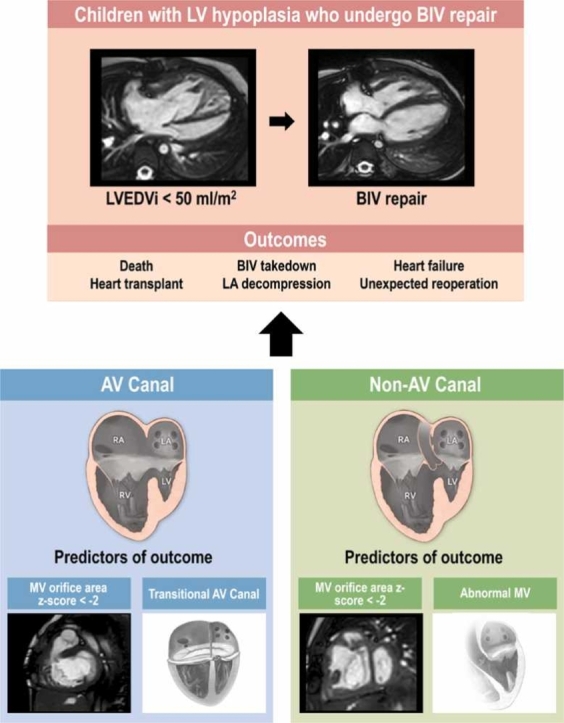

## 1 Introduction

Although staged palliation leading to the Fontan operation provides a path to survival for children with single ventricle physiology, the incremental improvements in ventricular volume load and cyanosis come at the expense of systemic venous hypertension [1]. As a result of the abnormal physiology state, the Fontan circulation is associated with a high incidence of debilitating and often life-threatening complications over time [2], [3], [4], [5]. Consequently, in patients who have a hypoplastic left ventricle (HLV), there is an increasing interest in offering an alternative approach aimed at a biventricular (BiV) circulation, with or without interim staged procedures to recruit/grow left heart structures [6], [7].

In patients with HLV who have undergone prior single ventricle palliation, a BiV repair represents a major change in circulation, reducing systemic venous pressure at the expense of increased pressure and/or volume load to the mitral valve (MV) and left ventricle. While many patients have favorable outcomes following BiV repair, a subset develop significant morbidity/mortality including MV dysfunction and left ventricular (LV) systolic and/or diastolic failure [8], [9], [10]. Although accurate patient selection is paramount to improve postoperative outcomes, risk prediction remains challenging, likely due to the complexity of pathology associated with HLV.

Prior echocardiographic and cardiovascular magnetic resonance (CMR) studies have found multiple associations with successful BiV repair, including larger MV size, larger LV size, and larger left to right atrioventricular (AV) valve, and ventricular volume ratios [11], [12], [13], [14], [15]. Endocardial fibroelastosis (EFE) and elevated LV filling pressure, surrogates for diastolic dysfunction/restrictive physiology, have also been shown to be important [16], [17]. While EFE is unambiguously one of the strongest risk factors, outcome predictors in diagnostic groups that lack EFE and patients with complex anatomical features, such as conotruncal anomalies and/or heterotaxy syndrome, have not been rigorously studied. Therefore, the aim of our study was to evaluate potential factors for association with adverse outcomes in a cohort of patients with HLVs without EFE. We chose to specifically focus on MV size and anatomy because of the wide range of MV pathology, as well as the limitations in surgical techniques to effectively repair the MV. [18] We also chose to limit our patient cohort to those with a preoperative CMR study because this modality is commonly used to evaluate patients and provides reliable data on valve morphology, and ventricular size and function. We hypothesized that a small MV orifice area would predict early failure after BiV conversion.

## 2 Methods

This was a single-center, retrospective cohort study. The Boston Children’s Hospital Institutional Review Board approved the study and informed consent was waived.

### 2.1 Study population

All patients with HLV and at least one available CMR scan prior to surgical BiV repair between 2005 and 2022 were screened for eligibility. We included patients with heterotaxy syndrome, right dominant atrioventricular canal (AVC) defects, and conotruncal lesions among others. Although patients may have undergone LV recruitment operations prior to BiV repair, we only included those with a pre-operative LV end-diastolic volume index of ≤50 mL/m^2^. Patients with EFE were excluded, as were patients with unmeasurable MV orifice area on pre-operative CMR imaging. Demographic, clinical information, CMR data, and surgical details were extracted from electronic medical records. When multiple CMR studies were available, the pre-operative study closest to the BiV repair surgery was included. Patients were divided into two anatomic sub-groups for analysis: 1) patients with a right dominant AVC with single AV vale annulus (AVC group) and 2) patients with HLV and two separate AV valve annuli (non-AVC group). This approach was chosen to account for the significant difference in AV valve morphology and surgical repair strategy between the two groups [18]. Complete AVC was defined as a primum atrial septal defect, AVC type ventricular septal defect (VSD), and the presence of a common AV valve. Transitional AVC was defined as a primum atrial septal defect and AVC type VSD with dense chordal attachments from the AV valve to the ventricular septum leading to insignificant ventricular level shunting. A partial AVC was defined as a primum atrial septal defect with no ventricular level shunting. All patients with complete, transitional, and partial AVC were included in the AVC group.

### 2.2 CMR

**Fig. 1:**
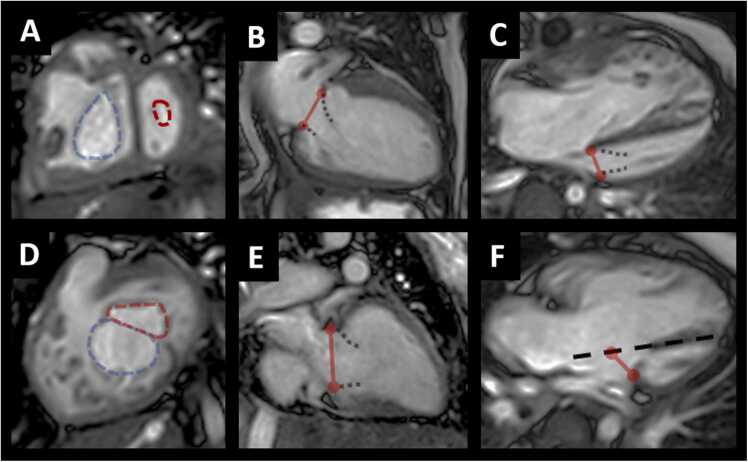
CMR measurements for non-atrioventricular canal (non-AVC) patients (A, B, C) and AVC patients (D, E, F). (A) Mitral and tricuspid valve orifice areas measured at end-diastole. (B) Mitral valve annulus diameter (2-chamber view) with leaflet measurements. (C) Mitral valve annulus diameter (4-chamber view). (D) Mitral valve orifice area was measured by dividing common atrioventricular valve along the plane of the intraventricular septum. (E) Mitral valve annulus diameter (2-chamber view) with leaflet measurements. (F) Mitral valve annulus diameter (4-chamber view).

Pre-operative CMR scans were performed at Boston Children’s Hospital using a 1.5T scanner (Philips Achieva, Philips Medical Systems, Best, The Netherlands). A typical imaging protocol included cine steady-state free precession imaging in axial and ventricular long- and short-axis planes. Typically, 10–12 short-axis images were acquired with a slice thickness of 8 mm, typical spatial resolution of 1.7–2 mm × 1.7–2 mm, and temporal resolution of 30–40 ms with 30 reconstructed phases per cardiac cycle. Cardiac measurements included ventricular volumes, ejection fraction, and flow data. Using commercially available post-processing software (Circle Cardiovascular Imaging Inc, cvi42, Alberta, Canada), MV anatomy and function were evaluated. The smallest MV orifice area was measured in a short-axis plane at the level of the MV leaflet tips during maximum opening of the MV at end-diastole. The leaflet tips were measured by finding the most apical slice where the MV leaflet tissue is visible and the cross-sectional area is measurable. Preoperative MV or common AV valve regurgitation was defined as none (0–5%), mild (6–20%), moderate (21–40%), and severe (>40%). Supplementary preoperative echocardiographic inflow gradients were used to define MV stenosis as none (0–3 mm Hg), mild (4–7 mm Hg), and moderate or greater (≥8 mm Hg). LV papillary muscle architecture was assessed and classified qualitatively as normal, closely spaced, or as a single papillary muscle. Valve morphology was also evaluated for congenital variants. For patients with a common AV valve, the size of the mural leaflet was classified as adequate or small/absent. Parachute MV was defined as all mitral or left AV valve chordal attachments to a single LV papillary muscle, for both AVC and non-AVC patients. Valve mechanics were also evaluated for restricted leaflet motion and leaflet prolapse. Patients with any abnormal feature of the MV (e.g., parachute valve, closely spaced papillary muscles, double orifice MV) were classified as having “abnormal MV anatomy.” The smallest MV orifice area was measured in a short-axis plane at the level of the MV leaflet tips during maximum opening of the MV at end-diastole. The MV orifice area in patients with an AVC was derived by extrapolating the plane of the ventricular septum through the common AV valve (Fig. 1). Similarly, MV annulus dimension in the 4-chamber view was obtained by extrapolating the plane of the ventricular septum through the common AV valve. Anterior and posterior MV leaflet lengths in non-AVC patents, and superior and inferior bridging leaflet lengths in AVC patients were measured when possible. Leaflet measurements were completed in the 2-chamber view at end-diastole from the distal part of the leaflet to its insertion point. Values for MV orifice area, 4-chamber annulus diameter, and 2-chamber annulus diameter were inputted into the Boston Children’s Hospital echo-derived z-score calculator (https://zscore.chboston.org/) to obtain normalized values [19], [20]. Note that z-scores for MV annulus-derived cross-sectional area were used to index the MV orifice area, as z-scores specific to MV orifice area were not available.

Interobserver and intraobserver reliability was assessed for a subset of CMR measurements of the MV using a random set of 40 patients (20 AVC, 20 non-AVC) by two separate observers.

### 2.3 Outcomes

The primary composite outcome was defined as time to all-cause mortality, listing for heart transplantation, BiV circulation takedown, heart failure admission, catheterization intervention for left atrial decompression, or unexpected reoperation. Reoperations for right ventricle-to-pulmonary artery conduit exchange were not included in the primary composite outcome as these may be expected operations based on the surgery performed. Reoperations for permanent pacemaker placement were not included in the composite outcome as the study cohort included patients with complex segmental anatomy and heterotaxy syndrome who were counseled regarding a risk of post-operative heart block. A secondary composite outcome was defined as moderate or greater mitral regurgitation and/or stenosis by post-operative imaging. Postoperative mitral regurgitation was defined as qualitatively moderate or greater by echocardiogram, or by regurgitation fraction ≥20% on CMR. Moderate mitral stenosis was defined as a mean gradient ≥8 mm Hg. For time-to-event analysis, follow-up was measured from the date of the BiV repair surgery to the composite outcome using the earliest outcome event in the case of multiple qualifying events. In cases without a qualifying event, follow-up time ended on date of last contact.

### 2.4 Statistical analysis

Data are presented as a mean with standard deviation unless the distribution was skewed, in which case median with interquartile range (IQR) is presented. Differences by AVC and non-AVC group were compared using independent samples t-test, Wilcoxon rank sum test, or Fisher exact test, as appropriate. Kaplan-Meier methodology was used to estimate the distribution of time to the primary outcome. The log-rank test was used to compare the distributions by group. The association of all variables with time to the primary outcome was examined using Cox proportional hazards regression. A multivariable Cox regression model was constructed to identify factors that were independently associated with the primary composite outcome by employing stepwise selection that included as candidates all factors with a univariate p-value less than 0.15. The criterion for remaining in the model was p < 0.05. A secondary analysis was conducted using the same methodology to determine the relationship of MV orifice area with the secondary outcome, presence of moderate or greater mitral stenosis or mitral regurgitation. An intra-class correlation (ICC) was estimated to measure agreement between CMR measurements among two observers, with 1 being perfect agreement and 0 being no agreement. A p-value of less than 0.05 was considered statistically significant. Analyses were performed using SAS version 9.4 (SAS Institute, Cary, North Carolina) and R version 4.03 (The R Foundation, Vienna, Austria) [21].

## 3 Results

### 3.1 Patient characteristics

Between 2005 and 2022, 122 patients (59 (48%) AVC defects and 63 (52%) non-AVC defects), age 3 ± 2.8 years at the time of BiV repair, met inclusion criteria. Fifty (41%) patients had heterotaxy syndrome, and an additional 15 (12%) patients had another genetic syndrome (Table 1). The AVC patients had a lower weight and body surface area compared with non-AVC patients. Otherwise, there were no demographic differences between the AVC and non-AVC groups. AVC patients were more likely to have heterotaxy syndrome (57% vs 27%), and less likely to have membranous, muscular, conoventricular, and multiple VSDs compared to non-AVC patients. Aside from VSD type, MV papillary muscle structure was the only significant cardiac anatomic difference between AVC and non-AVC patients. AVC patients were more likely to have a parachute MV and more likely to have AV valve regurgitation compared to non-AVC patients. Most (111; 89%) patients had undergone single ventricle palliation surgery prior to BiV repair, and 31 (25%) had a prior LV recruitment operation for LV growth (e.g., a Blalock-Taussig-Thomas shunt in a patient with a bidirectional Glenn shunt and/or a restriction of the atrial septum to direct more pulmonary venous return into the left ventricle). There were no differences in the type of prior palliative interventions between AVC and non-AVC patients, including prior MV surgery.

**Table 1:** Clinical characteristics at time of BiV repair.

	All (N = 122)	AV canal (N = 59)	Non-AV canal (N = 63)	p value
Age (years)	3 ± 2.8	2.7 ± 2.3	3.4 ± 3.1	0.18
Male	65 (53)	29 (49)	37 (56)	0.47
Weight (kg)	12.8 ± 7.2	11.1 ± 4.8	14.4 ± 8.6	**0.01**
Body surface area (m^2^)	0.6 ± 0.2	0.5 ± 0.2	0.6 ± 0.2	**0.01**
Race				0.77
White	65 (54)	34 (58)	31 (50)	
Black	5 (4)	3 (5)	2 (3)	
Asian	9 (7)	4 (7)	5 (8)	
Other/unavailable	42 (35)	18 (31)	24 (39)	
Hispanic or Latino	8 (9)	4 (9)	4 (9)	1.00
Heterotaxy syndrome	50 (41)	33 (57)	17 (27)	**<0.01**
Genetic syndrome*	15 (12)	8 (14)	7 (11)	0.79
Anatomy				
Complete AV canal	54 (44)	54 (92)	0 (0)	**<0.01**
Partial AV canal	2 (2)	2 (3)	0 (0)	0.23
Transitional AV canal	3 (3)	3 (5)	0 (0)	0.11
Left sided obstructive lesion(s)	39 (32)	17 (29)	22 (35)	0.56
Right sided obstructive lesion(s)	54 (44)	25 (42)	29 (46)	0.72
Double outlet right ventricle	52 (43)	25 (42)	27 (43)	1.00
Transposition of the great arteries	16 (13)	5 (9)	11 (18)	0.18
Tetralogy of Fallot	3 (3)	0 (0)	3 (5)	0.25
VSD type				
Membranous	16 (13)	2 (3)	14 (22)	**<0.01**
Muscular	30 (25)	9 (15)	21 (33)	**0.02**
AV canal type	71 (58)	55 (93)	16 (25)	**<0.01**
Cono-ventricular	34 (28)	3 (5)	31 (49)	**<0.01**
Conal-septal	3 (3)	0 (0)	3 (5)	0.25
Multiple VSDs	35 (29)	10 (17)	25 (40)	**<0.01**
Mitral/left AV valve anatomy				
Normal	27 (22)	0 (0)	27 (43)	**<0.01**
Closely spaced papillary muscles	45 (36)	26 (44)	19 (30)	0.14
Parachute	20 (16)	16 (27)	4 (6)	**<0.01**
Straddling	8 (7)	1 (2)	7 (11)	0.06
Shortened chordae	9 (7)	7 (12)	2 (3)	0.09
Double orifice mitral valve	4 (3)	3 (5)	1 (2)	0.40
Mitral valve inflow gradient (mm Hg)	0.6 ± 1.6	0.7 ± 1.7	0.5 ± 1.4	0.43
Mitral stenosis				0.63
None	105 (86)	52 (88)	53 (84)	
Mild	15 (12)	7 (12)	8 (13)	
Moderate	2 (2)	0 (0)	2 (3)	
Mitral or common AV valve regurgitation				**<0.01**
None	75 (62)	20 (34)	55 (87)	
Mild	30 (25)	23 (39)	7 (11)	
Moderate	12 (10)	11 (19)	1 (2)	
Severe	5 (4)	5 (9)	0 (0)	
Apex forming left ventricle	107 (88)	50 (85)	57 (91)	0.41
Prior palliative interventions				0.98
None	13 (11)	7 (12)	6 (10)	
Fontan	6 (5)	2 (3)	4 (6)	
Norwood	4 (3)	1 (2)	3 (5)	
Hybrid/stage 1	3 (3)	1 (2)	2 (3)	
Bidirectional Glenn shunt	48 (39)	23 (39)	25 (40)	
Shunted Glenn	11 (9)	5 (9)	6 (10)	
PA band	20 (16)	11 (19)	9 (14)	
BTT shunt	8 (7)	4 (7)	4 (6)	
Other	9 (7)	5 (9)	4 (6)	
Prior surgery to increase L heart flow	31 (25)	16 (27)	15 (24)	0.68
MV surgery prior to BiV repair	22 (18)	14 (24)	8 (13)	0.16
Pulmonary vein stenosis	12 (10)	7 (12)	5 (8)	0.55

Data presented as mean ± standard deviation or number (%).

Bold font indicates significant p value <0.05.

*AV* atrioventricular, *VSD* ventricular septal defect, *PA* pulmonary artery, *BTT* Blalock-Tausig-Thomas, *L* left, *MV* mitral valve, *BiV* biventricular.

* Excludes heterotaxy syndrome.

By CMR, the average LV end-diastolic volume prior to BiV repair was 41 ± 9 mL/m^2^, the average LV:RV stroke volume ratio was 0.55 ± 0.27, and 20 patients (16%) had a MV orifice area z-score <−2 (Table 2). Notable differences in CMR characteristics between AVC and non-AVC groups included a higher right ventricular end-diastolic volume index, lower LV:RV stroke volume ratio, and higher MV orifice area z-score in the AVC group. Leaflet lengths and AV valve diameters were measured but omitted from Table 2 as no significant associations were identified with the primary composite outcome.

**Table 2:** CMR data prior to BiV repair.

	All (N = 122)	AV canal (N = 59)	Non-AV canal (N = 63)	p value
Age (years)	2.8 ± 2.7	2.5 ± 2.3	3.1 ± 3.1	0.24
BSA	0.5 ± 0.2	0.5 ± 0.2	0.6 ± 0.2	0.05
Heart rate (beats/minute)	105 ± 20	105 ± 18	104 ± 22	0.68
LV EDVi (mL/m^2^)	40.8 ± 9.1	39.7 ± 9	41.8 ± 9.2	0.20
LV ESVi (mL/m^2^)	16.6 ± 4.9	16.6 ± 5	16.6 ± 4.9	0.97
LV SVi (mL/m^2^)	24.7 ± 6	23.7 ± 5.8	25.6 ± 6.2	0.08
LV EF (%)	59.8 ± 6.3	58.6 ± 6.1	61 ± 6.3	**0.04**
RV EDVi (mL/m^2^)	95.5 ± 38.9	105.3 ± 44.9	86.3 ± 29.8	**0.01**
RV ESVi (mL/m^2^)	42.7 ± 19.4	46.1 ± 21.5	39.5 ± 16.8	0.06
RV SVi (mL/m^2^)	52.9 ± 22.9	59.1 ± 27.1	47.3 ± 16.5	**<0.01**
RV EF (%)	55.8 ± 7	56.4 ± 7	55.4 ± 7.1	0.43
LV:RV stroke volume ratio	0.50 (0.3, 0.7)	0.4 (0.3, 0.7)	0.6 (0.4, 0.7)	**<0.01**
MV:TV inflow ratio*	0.7 (0.4, 0.9)	0.7 (0.3, 1)	0.7 (0.5, 0.8)	0.71
MV orifice area	1.5 ± 0.6	1.5 ± 0.5	1.4 ± 0.7	0.36
MV orifice area (z-score)	−0.8 ± 1.3	−0.4 ± 1.3	−1.2 ± 1.3	**<0.01**
MV orifice area z-score <−2 (total N)	20 (16.4%)	4 (6.8%)	16 (25.4%)	**<0.01**
MV annulus diameter (4Ch view, z-score)	−2.2 ± 2.1	−2.8 ± 2.3	−1.7 ± 1.8	**<0.01**
MV annulus diameter (2Ch view, z-score)	0.5 ± 2.4	1.4 ± 2.8	−0.1 ± 1.8	**<0.01**
MV:TV cross-sectional area	0.6 (0.4, 0.8)	0.6 (0.4, 0.8)	0.6 (0.4, 0.8)	0.90
Total AVV cross-sectional area (cm^2^)	3.9 ± 1.4	4.2 ± 1.4	3.6 ± 1.2	**0.03**

Data presented as mean ± standard deviation or median (IQR).

Bold font indicates significant p value <0.05.

*CMR* cardiovascular magnetic resonance, *BiV* biventricular, *IQR* interquartile range, *BSA* body surface area, *LV* left ventricle, *EDVi* end diastolic volume index, *ESVi* end systolic volume index, *SVi* stroke volume index, *EF* ejection fraction, *RV* right ventricle, *MV* mitral valve, *TV* tricuspid valve, *AVV* atrioventricular valve, *4Ch* four chamber, *2Ch* two chamber

* Inflow data not available for multiple patients.

### 3.2 Outcome analysis

**Fig. 2:**
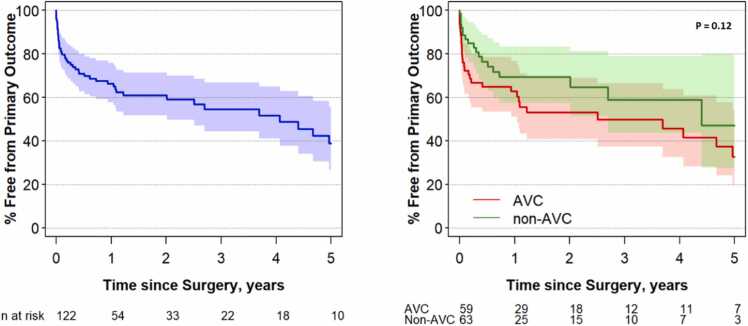
Estimated freedom from the primary composite outcome for the overall cohort (**left**) and cohort stratified by atrioventricular canal (AVC) and non-AVC status (**right**) with 95% confidence bands (p = 0.12). The primary composite outcome included time to death, transplant, biventricular takedown, heart failure admission, left atrial decompression, or unexpected reoperation. The median time to the primary composite outcome (time by which 50% of patients had experienced the primary outcome) was 2.7 years overall (2.5 years in AVC group and 4.4 years in the non-AVC group). At 2 years, the event-free survival was 53% vs 69% for the AVC and non-AVC groups, respectively.

At a median (IQR) follow-up of 0.7 (0.1, 2.2) years, 49 (40%) of patients met the primary composite outcome measure (44 unexpected reoperations, 11 deaths, 6 heart failure admissions, 4 cardiac catheterizations for left atrial decompression, 1 transplant listing, and 1 BiV take-down). The median time to the composite outcome was 2.7 years overall (2.5 years in AVC group and 4.4 years in the non-AVC group; p = 0.12). The frequencies of outcome according to earliest occurring event were unexpected reoperation (n = 42), death (n = 3), BiV takedown (n = 1), and heart failure admission (n = 3). Twenty-three patients returned to the operating room for either an MV repair or replacement. Other common re-operations included post-operative extracorporal membrane oxygenation (ECMO) cannulation (n = 10), relief of sub-aortic obstruction (n = 8), and residual VSD closure (n = 9) (Supplemental Table 1). Freedom from the primary outcome at 2 years was 53% and 69% for the AVC and non-AVC groups (log rank p = 0.12) (Fig. 2).

By univariate analysis, transitional AVC defect at the BiV repair was associated with the primary composite outcome in AVC patients. In non-AVC patients, MV orifice area z-score <−2, abnormal MV anatomy, mild or greater preoperative mitral stenosis, and larger RV end-systolic volume index were all associated with the primary composite outcome. In multi-variable Cox regression analysis, MV orifice area z-score <−2 and transitional AVC classification were associated with the primary outcome in the AVC group. Similar analysis in the non-AVC group demonstrated an independent association with MV orifice area z-score <−2, abnormal MV anatomy, and conal septal VSD (Table 3).

**Table 3:** Univariate and multivariable Cox regression for time to primary outcome in AV canal and non-AV canal patients (N = 122).

	AV canal (N = 59)				Non-AV canal (N = 63)			
	Univariate		Multivariable		Univariate		Multivariable	
	HR (95% CI)	p	HR	p	HR (95% CI)	p	HR	p
Age at surgery (years)	1 (0.8, 1.1)	0.57			0.9 (0.7, 1.1)	0.24		
Male	0.8 (0.4, 1.7)	0.56			1.3 (0.5, 3.2)	0.62		
Weight at surgery, kg	1 (0.9, 1.1)	0.85			1 (0.9, 1)	0.26		
BSA at surgery, m^2^	0.7 (0.1, 6.4)	0.76			0.2 (0, 2.2)	0.19		
Race		0.80				0.91		
White	1.4 (0.2, 10.9)				0.8 (0.1, 6)			
Black	Ref				Ref			
Asian	1.1 (0.1, 12.4)				0 (0, NA)			
Other	1.9 (0.3, 15.3)				0.5 (0.1, 4.6)			
Hispanic	0 (0, NA)	0.99			1.6 (0.4, 7.3)	0.54		
Non-cardiac comorbidity	1.4 (0.6, 3.5)	0.46			3.4 (1, 11.9)	0.05		
Heterotaxy	0.8 (0.4, 1.7)	0.58			0.9 (0.3, 2.5)	0.83		
Genetic syndrome	0.3 (0.1, 1.5)	0.15			2.0 (0.6, 6.9)	0.28		
Anatomy								
Complete AV canal	0.4 (0.2, 1.3)	0.12			-	n/a		
Partial AV canal	0.8 (0.1, 6)	0.84			-	n/a		
Transitional AV canal	**5.4 (1.5, 19.4)**	**<0.01**	**6.2 (1.7, 22.4)**	**0.01**	-	n/a		
Left-sided obstructive lesion(s)	1.2 (0.6, 2.5)	0.66			2.1 (0.8, 5.1)	0.12		
Right-sided obstructive lesion(s)	1.1 (0.6, 2.3)	0.73			**0.3 (0.1, 0.9)**	**0.03**		
Double outlet right ventricle	0.8 (0.4, 1.6)	0.48			0.5 (0.2, 1.4)	0.19		
Transposition of the great arteries	1.2 (0.4, 4.1)	0.74			1.5 (0.4, 5.3)	0.53		
Tetralogy of Fallot	-	n/a			0.9 (0.1, 6.8)	0.93		
VSD type								
Membranous	1.1 (0.2, 8)	0.94			1.7 (0.6, 4.4)	0.30		
Muscular	0.4 (0.1, 1.8)	0.25			0.9 (0.3, 2.3)	0.76		
AV-canal type	1 (0.2, 4.4)	0.96			0.9 (0.3, 2.6)	0.78		
Conoventricular	2 (0.5, 8.4)	0.36			0.5 (0.2, 1.3)	0.18		
Conal-septal	-	n/a			0.6 (0.3, 1.2)	0.15	**6.1 (1.6, 23.4)**	**<0.01**
Multiple	0.7 (0.2, 2.3)	0.55			1.1 (0.5, 2.8)	0.79		
Mitral/left AV valve								
Abnormal anatomy	-	n/a			**7.9 (1.8–34.5)**	**<0.01**	**2.9 (1.1, 7.8)**	**0.01**
Closely spaced papillary muscles	1.3 (0.7, 2.7)	0.43			**3.8 (1.5, 9.7)**	**<0.01**		
Parachute	0.8 (0.4, 1.8)	0.59			1.5 (0.3, 6.4)	0.63		
Straddling	0 (0, NA)	n/a			1.3 (0.4, 4.6)	0.66		
Shortened chordae	1.7 (0.6, 5)	0.35			2.2 (0.3, 16.3)	0.46		
Double orifice mitral valve	1.4 (0.3, 6.1)	0.62			0 (0, NA)	n/a		
Mitral stenosis (≥ mild)	2.3 (0.9, 6.2)	0.09			**3.5 (1.3, 9.6)**	**0.02**		
Mitral regurgitation (≥ mild)	1 (0.5, 2.2)	0.94			1.6 (0.5, 4.7)	0.44		
Apex forming left ventricle	0.9 (0.4, 2.4)	0.86			1 (0.2, 4.5)	0.99		
Prior palliative interventions		0.25				0.82		
None	Ref				Ref			
Fontan	0.9 (0.2, 5.1)				1.6 (0.1, 26.2)			
Norwood	4 (0.7, 22.9)				1.7 (0.1, 28.9)			
Hybrid/stage 1 palliation	0 (0, NA)				4.8 (0.4, 53.3)			
Bidirectional Glenn shunt	8.6 (0.9, 86.3)				0 (0, NA)			
Shunted Glenn	1 (0.3, 3)				2.4 (0.3, 21)			
PA band	0.6 (0.1, 3)				5 (0.5, 51.4)			
BTT shunt	0.4 (0.1, 1.9)				5 (0.5, 47.7)			
Other	1.2 (0.3, 5)				2 (0.1, 31.9)			
Prior procedure to inc. L heart flow	1.1 (0.5, 2.4)	0.79			1.2 (0.5, 3.1)	0.73		
MV surgery prior to BiV	1 (0.5, 2.3)	0.95			1.8 (0.6, 5.7)	0.30		
CMR parameters								
LV EDVi (mL/m^2^)	1 (1, 1.02)	0.46			1 (0.9, 1)	0.29		
LV ESVi (mL/m^2^)	1 (0.9, 1)	0.33			1 (0.9, 1.1)	0.49		
LV SVi (mL/m^2^)	1 (1, 1.1)	0.78			1 (0.9, 1)	0.18		
LV EF (%)	1 (1, 1.1)	0.54			1 (0.9, 1.1)	0.80		
RV EDVi (mL/m^2^)	1 (1, 1)	0.50			1 (1, 1)	0.16		
RV ESVi (mL/m^2^)	1 (1, 1)	0.98			**1 (1, 1)**	**0.04**		
RV SVi (mL/m^2^)	1 (1, 1)	0.27			1 (1, 1)	0.57		
RV EF (%)	1 (0.9, 1)	0.50			1 (0.9, 1)	0.15		
LV:RV stroke volume ratio	1.4 (0.3, 6.7)	0.71			0.2 (0, 1.2)	0.07		
MV:TV inflow ratio	0.7 (0.4, 0.9)	0.79			0.7 (0.6, 0.8)	0.98		
MV orifice area (cm^2^)	0.8 (0.4, 1.5)	0.46			0.6 (0.3, 1.2)	0.14		
MV orifice area z-score	0.9 (0.7, 1.2)	0.49			0.8 (0.6, 1.2)	0.31		
MV orifice area z < −2	2.7 (0.9, 7.8)	0.07	**3 (1, 8.9)**	**0.04**	**2.9 (1.1, 7.5)**	**0.03**	**3.9 (1.4, 11)**	**0.01**
MV annulus (4C z-score)	0.9 (0.8, 1.1)	0.42			0.9 (0.7, 1.2)	0.49		
MV annulus (2C z-score)	1.1 (1, 1.2)	0.26			0.9 (0.7, 1.2)	0.61		
MV:TV area, N = 59/61	0.7 (0.2, 2.5)	0.57			0.4 (0.1, 2.1)	0.25		
Total AVV area (cm^2^)	0.9 (0.7, 1.2)	0.50			0.7 (0.5, 1.1)	0.14		

Data presented as hazard ratio (95% confidence interval) and p-value.

Bold font indicates significant p value <0.05

*AV* atrioventricular, *VSD* ventricular septal defect, *PA* pulmonary artery, *BTT* Blalock-Tausig-Thomas, *L* left, *MV* mitral valve, *LV* left ventricle, *EDVi* end diastolic volume index, *ESVi* end systolic volume index, *SVi* stroke volume index, *EF* ejection fraction, *RV* right ventricle, *AVV* atrioventricular valve.

**Fig. 3:**
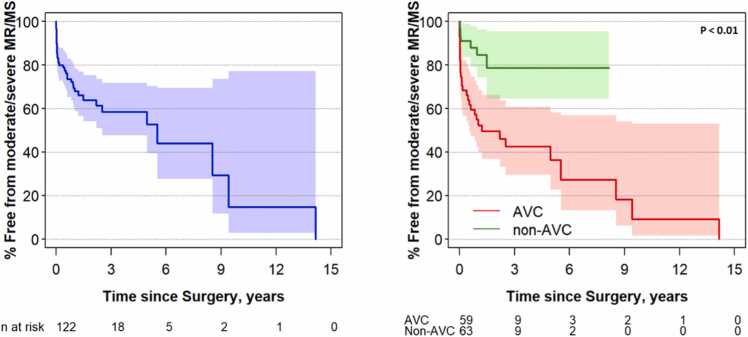
Estimated freedom from the secondary composite outcome for the overall cohort (**left**) and cohort stratified by atrioventricular canal (AVC) and non-AVC status (**right**) with 95% confidence bands (p < 0.01). At 2 years, the event-free survival was 49% vs 79% for the AVC and non-AVC groups, respectively. *MR* mitral regurgitation*, MS* mitral stenosis.

Overall, 40/122 (33%) patients met the secondary outcome (28 with more than or equal to moderate mitral regurgitation, 7 with more than or equal to moderate mitral stenosis, and 5 with more than or equal to moderate mitral regurgitation and mitral stenosis). Freedom from the secondary outcome at 2 years was 49% and 79% for the AVC and non-AVC groups respectively (log rank p < 0.01) (Fig. 3). For AVC patients, transitional AVC morphology was associated with higher risk of the secondary outcome, and common AV valve division at the time of the BiV repair was associated with lower risk of the secondary outcome (Supplemental Table 2). For the non-AVC group, multiple associations were identified including left heart obstructive lesions, MV pathology (parachute valve, more than or equal to mild preoperative mitral stenosis), a history of mitral valvuloplasty prior to BiV repair, and a membranous VSD.

By multivariable analysis, older age at time of surgery, transitional AVC defect, and transposition of the great arteries were independently associated with the secondary outcome for AVC patients. Multivariable Cox modeling was not possible in the non-AVC group due to the small number of secondary outcome events.

### 3.3 Intraobserver and interobserver variability

In the AVC group, the mean intraobserver differences were −0.01 ± 0.12 mm for MV orifice area with an ICC of 0.94; and interobserver differences were 0.00 ± 0.06 mm with an ICC of 0.99. In the non-AVC group, the mean intraobserver differences were −0.02 ± 0.08 mm for MV orifice area with an ICC of 0.99; and interobserver differences were −0.01 ± 0.08 mm with an ICC of 0.94.

## 4 Discussion

We studied 122 preoperative CMR examinations in patients with HLV (LV end-diastolic volume ≤50 mL/m^2^) to assess for risk factors prior to BiV repair. We found multiple notable factors related to the MV that were associated with worse outcomes, including MV orifice area below a threshold z-score of −2, transitional AVC sub-type, and other MV anatomical features, such as closely spaced papillary muscles and parachute MV morphology. Although there was no significant difference in the composite primary outcome between AVC and non-AVC groups, the AVC group had a higher risk of postoperative MV dysfunction in secondary analysis. Our data thus provide salient and widely applicable information for assessment of BiV repair risk in this increasingly complex and vulnerable population.

Prior work has shown that MV size (including annulus size and common AV valve area) is a predictor of survival after BiV repair in HLV patients; however, studies were limited to echocardiographic images and also excluded patients with heterotaxy syndrome and complex intracardiac anatomy [12], [13], [14], [15]. Interestingly, we found that neither 4-chamber nor 2-chamber MV annulus z-score was predictive of either primary or secondary outcome for both AVC and non-AVC patients based on our CMR measurements nor was total AV valve area predictive in either group. Aside from annulus size, anatomic features of the MV with a potential to limit flow also include abnormal leaflet, chordal, and papillary muscle architecture that serve to restrict leaflet motion [22]. Because MV adequacy cannot be reduced to annulus measurements alone, we measured the smallest MV cross-sectional area at end-diastole by CMR planimetry, as this is known to have excellent correlation with effective orifice area and may be a better predictor of outcomes in this patient population [23]. Our finding that an MV orifice area z-score <−2 was associated with a higher risk of primary outcome in both AVC and non-AVC patients suggests that MV orifice area should be a major consideration when contemplating BiV repair in HLV patients. Furthermore, the lack of an association between preoperative mitral stenosis severity (based on echocardiogram gradients) and outcome supports our assertion that anatomic factors are more useful in the preoperative evaluation of this patient cohort. Although preoperative MV gradients are important data, they must be taken in context of the underlying physiology and volume of flow across the MV.

Our study builds on prior work by identifying transitional AVC as a subtype with worse outcome in right dominant AVC patients. Transitional AVC was not only an independent risk factor for the primary outcome but was also associated with worse post-operative MV function in the secondary analysis. All three patients in our study with a transitional AVC returned to the operating room after BiV repair for an MV repair or replacement, and one patient required relief of LV outflow tract obstruction. Despite re-operations, two of the three patients continue to have moderate mitral regurgitation. These findings are consistent with prior observations that patients with a transitional AVC have a high incidence of developing MV dysfunction and LV outflow tract obstruction following repair. This may be related to shortened leftward components of the bridging leaflets with abnormal anchoring to the ventricular septum, crowding the LV outflow tract and resulting increased tension after cleft closure [24], [25], [26], [27].

The lack of additional MV risk factors in patients with AVC defects likely speaks to the modified surgical approach to AV valve septation, in which the AV valve can be divided more evenly—by partitioning the VSD patch toward the right side of the common AV valve—to create a larger MV orifice [27]. In patients with two separate AV valve orifices (partial/transitional AVC and non-AVC patients), surgical options to repair a hypoplastic MV are often limited by small space and less opportunity to compensate for abnormal native anatomy [28]. These findings support our overall hypothesis that pre-existing MV pathology at the time of BiV repair (closely spaced papillary muscles, parachute MV, mild or greater stenosis, and a history of mitral valvuloplasty) is predictive of future MV dysfunction but appears to be more relevant to patients with two separate AV valves. It is worth noting that while additional MV risk factors were not identified among the AVC group (other than MV orifice area), patients with AVC had higher incidence of post-operative MV dysfunction and therefore AVC status should also be considered a surgical risk factor.

Previous studies have demonstrated that higher CMR-derived LV end-diastolic volume index, mitral:tricuspid inflow ratio, and left ventricle:right ventricle stroke volume ratio predict survival after BiV repair [11]. Unexpectedly, we did not find any of these three parameters to be associated with either primary or secondary outcomes. One possible explanation is that our cohort represents a more contemporary pre-selected group, and patients with previously documented unfavorable CMR parameters did not undergo BiV repair. This is supported by the CMR imaging data in our study where very few patients had previously defined cutoff values of LV end diastolic volume index <30 mL/m^2^, mitral:tricuspid inflow ratio <0.4, or left ventricle:right ventricle stroke volume ratio <0.25.

In patients with HLV, the decision regarding whether to pursue a BiV repair vs Fontan operation can be challenging, particularly because each approach is typically considered a definitive strategy that cannot be easily “reversed.” It is worth noting that in our study, a significant proportion of patients had risk factors for Fontan failure (common AV valve regurgitation, MV regurgitation, pulmonary vein stenosis, and heterotaxy syndrome) and were thus considered poor Fontan candidates [17], [29]. While it is plausible that these same risk factors may have impacted their chances of a successful BiV repair strategy, none was associated with the primary or secondary outcomes in our study. Still, it should be emphasized that the decision to pursue a BiV repair should be individualized in the context of multiple patient-specific factors, such as MV size and anatomy, underlying diagnosis, ventricular size and function among others. Future studies comparing outcomes between risk-matched patients palliated with a Fontan circulation vs BiV repair are needed to further inform clinical decision-making.

## 5 Limitations

This study has several limitations. Requiring a pre-operative CMR for study inclusion likely led to selection of patients with more complex anatomy than the general population of patients with HLV. In addition, the study population only included patients who underwent a BiV conversion. Patients were excluded if plans for BiV conversion were aborted following direct surgical inspection; and therefore, the study is not an intention-to-treat analysis. The small sample size may have resulted in limited power to find significant associations with the primary or secondary composite outcomes. The precision in MV orifice measurements may have been limited by an average short-axis slice thickness of 8 mm; and future research investigating the optimal way to measure MV orifice area may be useful. MV severity determination by inflow gradients may be confounded by single ventricle physiology preoperatively, and the presence of cardiac shunt lesions or valve regurgitation postoperatively. Some patients had intentionally created atrial level left-to-right shunts during or after BiV repair, and this may have underestimated the number of patients with moderate or greater mitral stenosis. Many of the patients in the study were referred to our heart center for surgery and then returned to their home institution following surgical repair. This resulted in limited follow-up data and a generally short follow-up period. The shorter follow-up period for non-AVC patients could represent a lower rate of primary and secondary outcomes compared to AVC patients, as patients will often return to our institution for reoperations and thus allow follow-up status to be recorded.

## 6 Conclusions

We provide novel information regarding predictors of adverse outcomes following BiV repair for patients with an HLV without EFE. For both AVC and non-AVC patients, MV orifice area <−2 is associated with higher risk of time to death, transplant, BiV takedown, heart failure admission, left atrial decompression, or unexpected reoperation. Additional risk factors include transitional AVC morphology in AVC patients, and abnormal MV anatomy in non-AVC patients. Further studies are needed in this challenging patient population to further refine an optimal decision-making algorithm for a BiV repair.

## Funding

Not applicable.

## Author contributions

**David N. Schidlow:** Conceptualization, Writing – original draft, Writing – review and editing. **Eric Feins:** Conceptualization, Investigation, Writing – original draft, Writing – review and editing. **Minmin Lu:** Data curation, Formal analysis. **Lynn A. Sleeper:** Data curation, Formal analysis. **Addison Gearhart:** Data curation, Formal analysis, Writing – review and editing. **David Liddle:** Conceptualization, Data curation, Formal analysis, Investigation, Methodology, Validation, Writing – original draft, Writing – review and editing. **Rebecca S. Beroukhim:** Conceptualization, Data curation, Formal analysis, Investigation, Methodology, Supervision, Validation, Writing – original draft, Writing – review and editing. **Sitaram Emani:** Conceptualization, Writing – original draft, Writing – review and editing. **Andrew J. Powell:** Conceptualization, Writing – original draft, Writing – review and editing. **Sunil Ghelani:** Writing – original draft, Writing – review and editing.

All authors have approved the submitted version and have agreed both to be personally accountable for the author’s own contributions and to ensure that questions related to the accuracy or integrity of any part of the work, even ones in which the author was not personally involved, are appropriately investigated, resolved, and the resolution documented in the literature.

## Ethics approval and consent

The Boston Children’s Hospital Institutional Review board approved the study (IRB-P00039802) and informed consent was waived.

## Consent for publication

Not applicable.

## Declaration of competing interests

The authors declare that they have no known competing financial interests or personal relationships that could have appeared to influence the work reported in this paper.

## Footnotes

Appendix A Supplementary data associated with this article can be found in the online version at doi:10.1016/j.jocmr.2024.101029.
