# Supplementary material for: Mitral valve orifice area predicts outcome after biventricular repair in patients with hypoplastic left ventricles
Source: J Cardiovasc Magn Reson. 2024 Feb 23;26(1):101029. doi: 10.1016/j.jocmr.2024.101029 (PMC10965470; doi:10.1016/j.jocmr.2024.101029)
Supplement: Supplementary file 1 — Supplementary material [file mmc1.docx]

Supplemental Table 1. Expected and unexpected reoperations following BiV repair.

|  | All  N=122 | AV canal  N=59 | Non-AV canal  N=63 |
| --- | --- | --- | --- |
| All reoperations | 63 (52) | 38 (64) | 25 (4) |
| Unexpected reoperations | 44 (36) | 28 (47) | 16 (25 |
| Mitral valve repair/replacement | 23 (19) | 16 (27) | 7 (11) |
| ECMO | 10 (8) | 6 (10) | 4 (6) |
| Residual VSD closure | 9 (7) | 5 (8) | 4 (6) |
| Sub-aortic obstruction relief | 8 (7) | 6 (10) | 2 (3) |
| Tricuspid valve repair | 8 (7) | 4 (7) | 4 (6) |
| Aortic valve repair/replacement | 3 (2) | 1 (2) | 2 (3) |
| Emergent re-opening | 3 (2) | 1 (2) | 2 (3) |
| BiV takedown | 1 (1) | 1 (2) | 0 (0) |
| Expected reoperations | 19 (16) | 10 (17) | 9 (14) |
| Right ventricle-to-pulmonary artery conduit replacement | 9 (7) | 4 (7) | 5 (8) |
| Permanent pacemaker placement | 13 (11) | 7 (12) | 6 (10) |

Data presented as number (%).

Total number of patients requiring reoperation = 40/122 (33%). An individual patient may have had multiple reoperations.

Abbreviations: VSD = ventricular septal defect, BiV = biventricular, ECMO = extra corporeal membrane oxygenation.

Supplemental Table 2. Univariate and multivariable cox regression models for time to secondary outcome in AV canal and non-AV canal patients (N=122).

|  | **AV canal (N=59)** | | | | **Non-AV canal (N=63)** | |
| --- | --- | --- | --- | --- | --- | --- |
|  | **Univariate** | | **Multivariable** | | **Univariate** | |
|  | **HR (95% CI)** | **p** | **HR** | **p** | **HR (95%CI)** | **p** |
| Age (years) | 1.1 (1, 1.3) | 0.12 | **1.2 (1.03, 1.4)** | **0.02** | 0.9 (0.6, 1.2) | 0.33 |
| Weight at surgery, kg | 1.0 (1, 1.1) | 0.34 |  |  | 0.9 (0.8, 1.1) | 0.17 |
| Male | 0.6 (0.3, 1.3) | 0.20 |  |  | 0.7 (0.2, 2.9) | 0.63 |
| BSA at surgery, m^2^ | 2.8 (0.3, 29.6) | 0.40 |  |  | 0.03 (0, 2.6) | 0.13 |
| Race |  | 0.94 |  |  |  | 0.93 |
| White | Ref |  |  |  | Ref |  |
| Black | 0 (0, NA) |  |  |  | 0 (0, NA) |  |
| Asian | 1.4 (0.4, 4.6) |  |  |  | 0 (0, NA) |  |
| Other | 1.2 (0.5, 2.9) |  |  |  | 0.7 (0.1, 6.8) |  |
| Hispanic | 2.1 (0.6, 7.4) | 0.25 |  |  | 2.1 (0.2, 17.9) | 0.50 |
| Heterotaxy | 1.3 (0.6, 2.7) | 0.47 |  |  | 0.9 (0.2, 4.6) | 0.92 |
| Genetic syndrome* | - | n/a |  |  | - | n/a |
| Anatomy |  |  |  |  |  |  |
| Complete AV canal | 0.7 (0.2, 2.2) | 0.48 |  |  | - | n/a |
| Transitional AV canal | **5.5 (1.2, 25.0)** | **0.03** | **9.0 (1.7, 47.3)** | **<0.01** | - | n/a |
| Left sided obstructive lesion(s) | 1.2 (0.6, 2.5) | 0.63 |  |  | **7.3 (1.5, 36.6)** | **0.02** |
| Right sided obstructive lesion(s) | 0.9 (0.4, 1.8) | 0.66 |  |  | 0.3 (0.1, 1.4) | 0.13 |
| Double outlet right ventricle | 1.1 (0.5, 2.3) | 0.84 |  |  | 0.3 (0.1, 1.5) | 0.13 |
| Transposition of the great arteries | 2.5 (0.9, 7.4) | 0.09 | **4.2 (1.4, 12.9)** | **0.01** | - | n/a |
| VSD type |  |  |  |  |  |  |
| Membranous | 1.1 (0.2, 8.0) | 0.94 |  |  | **7.7 (1.8, 33.2)** | **<0.01** |
| Muscular | 0.4 (0.1, 1.5) | 0.16 |  |  | 2.0 (0.5, 8.2) | 0.31 |
| AV-canal type | 2.0 (0.5, 8.5) | 0.35 |  |  | 0.5 (0.1, 3.6) | 0.45 |
| Conoventricular | 0.4 (0.1, 3.2) | 0.4 |  |  | 0.1 (0, 1.0) | 0.05 |
| Conal-septal | - | n/a |  |  | - | n/a |
| Multiple | 0.4 (0.1, 1.7) | 0.22 |  |  | 1.8 (0.4, 7.1) | 0.43 |
| Mitral/left AV valve anatomy |  |  |  |  |  |  |
| Closely spaced papillary muscles | 1.6 (0.8, 3.4) | 0.23 |  |  | 1.0 (0.2, 4.7) | 0.96 |
| Parachute | 0.6 (0.3, 1.5) | 0.28 |  |  | **9.9 (2.3, 42)** | **<0.01** |
| Straddling | 2.3 (0.3, 17.5) | 0.41 |  |  | 0.9 (0.1, 7.7) | 0.96 |
| Shortened chordae | 2.4 (1.0, 6.1) | 0.06 |  |  | - | n/a |
| Double orifice mitral valve | 1.2 (0.3, 1.5) | 0.28 |  |  | - | n/a |
| Mitral stenosis (≥ mild) | 1.8 (0.6, 5.3) | 0.28 |  |  | **5.4 (1.2, 24.2)** | **0.03** |
| Mitral regurgitation (≥ mild) | 0.9 (0.4, 1.9) | 0.80 |  |  | 3.8 (0.9, 15.9) | 0.07 |
| Apex forming Left Ventricle | 1.1 (0.4, 3.0) | 0.82 |  |  | 0.8 (0.1, 6.7) | 0.84 |
| Prior procedure to increase L heart flow | 1.7 (0.8, 3.7) | 0.19 |  |  | 3.0 (0.8, 12.3) | 0.12 |
| MV surgery prior to BiV repair | 1.2 (0.5, 2.7) | 0.67 |  |  | **4.8 (1.1, 21.6)** | **0.04** |
| **CMR parameters** |  |  |  |  |  |  |
| LV EDVi (ml/ m^2^) | 1.0 (1.0, 1.1) | 0.22 |  |  | 1.0 (0.9, 1.1) | 0.64 |
| LV ESVi (ml/ m^2^) | 1.1 (1.0, 1.1) | 0.22 |  |  | 1.0 (0.9, 1.2) | 0.78 |
| LV SVi (ml/ m^2^), N=58/63 | 1.0 (1.0, 1.1) | 0.15 |  |  | 1.0 (0.9, 1.1) | 0.75 |
| LV EF (%) | 1.0 (0.9, 1.0) | 0.61 |  |  | 1.0 (0.9, 1.1) | 0.89 |
| RV EDVi (ml/ m^2^) | 1.0 (1.0, 1.0) | 0.37 |  |  | 1.0 (1.0, 1.0) | 0.08 |
| RV ESVi (ml/ m^2^) | 1.0 (1.0, 1.0) | 0.75 |  |  | 1.0 (1.0, 1.1) | 0.02 |
| RV SVi (ml/ m^2^), N=58/63 | 1.0 (1.0, 1.0) | 0.20 |  |  | 1.0 (1.0, 1.1) | 0.22 |
| RV EF (%) | 1.0 (0.9, 1.0) | 0.17 |  |  | 1.0 (0.9, 1.1) | 0.63 |
| LV:RV SV ratio, N=58/63 | 3.6 (0.9, 15.0) | 0.08 |  |  | 0.1 (0.0, 2.8) | 0.19 |
| MV:TV inflow ratio, N=20/50 | 1.4 (0.7, 2.6) | 0.37 |  |  | 1.1 (0.2, 5.4) | 0.93 |
| MV orifice area (cm^2^) | 1.1 (0.6, 2.2) | 0.77 |  |  | 0.6 (0.2, 1.8) | 0.4 |
| MV orifice area (z-score) | 0.9 (0.7, 1.2) | 0.64 |  |  | 1.0 (0.6, 1.7) | 0.9 |
| MV orifice area z-score < -2 | 2.1 (0.7, 6.2) | 0.16 |  |  | 2.9 (0.7, 12.4) | 0.16 |
| MV annulus (4C z-score) | 1.0 (0.8, 1.2) | 0.87 |  |  | 1.1 (0.8, 1.6) | 0.51 |
| MV annulus (2C z-score), N=43/63 | 1.0 (0.90, 1.2) | 0.77 |  |  | 1.2 (0.9, 1.8) | 0.28 |
| MV:TV area ratio, N=59/61 | 2.0 (0.7, 5.3) | 0.19 |  |  | 0.3 (0.02, 3.9) | 0.34 |
| Total AVV area ( cm^2^) | 0.9 (0.7, 1.2) | 0.47 |  |  | 0.8 (0.4, 1.4) | 0.37 |

*Excludes heterotaxy syndrome.

Abbreviations: kg kilogram, m^2^ meters squared, AV atrioventricular, VSD ventricular septal defect, PA pulmonary artery, BTT Blalock-Tausig-Thomas, L left, MV mitral valve, LV left ventricle, EDVi end diastolic volume index, ESVi end systolic volume index, SVi stroke volume index, EF ejection fraction, RV right ventricle, AVV atrioventricular valve.
